# Predictors of surgical site infection/fracture related infection after open tibial fracture

**DOI:** 10.1515/med-2026-1493

**Published:** 2026-09-02

**Authors:** Yabin Liu, Guoqi Ji, Chengkuo Cai, Hengsheng Shu

**Affiliations:** Second Department of Limb Trauma Orthopaedic and Reconstructive Surgery, Tianjin Hospital, Tianjin, China; First Department of Limb Trauma Orthopaedic and Reconstructive Surgery, Tianjin Hospital, Tianjin, China

**Keywords:** fracture-related infection, open tibial fracture, risk prediction, landmark model, elastic net, decision curve analysis

## Abstract

**Objectives:**

To develop and internally validate leakage-robust landmark models to estimate 12-month fracture-related infection (FRI) risk after open tibial fracture at admission and postoperative day 3 (POD3).

**Methods:**

In this single-center retrospective cohort, adults with open tibial fractures treated with limb salvage between January 2023 and June 2024 were included. Definite 12-month FRI was adjudicated using the international FRI consensus definition. Two landmark models were prespecified: admission and postoperative day 3 (POD3). Predictors included injury severity, contamination, host and physiologic factors, antibiotic timing, and, at POD3, early clinical course, routine laboratory results, debridement timing, and soft-tissue coverage status. Elastic net–penalized logistic regression with restricted cubic splines was used. Missing data were addressed with multiple imputation nested within 1,000-bootstrap internal validation. Performance was assessed by AUC, Brier score, calibration, and decision curve analysis.

**Results:**

Among 913 patients, 125 (13.7 %) developed FRI. The admission model achieved an AUC of 0.77 and Brier score of 0.097. The POD3 model improved performance (AUC 0.82; Brier 0.089) and showed slightly greater net benefit across 5–25 % thresholds.

**Conclusions:**

Landmark models provided internally validated 12-month FRI risk estimates at admission and POD3, with modest improvement after POD3 updating. External validation and recalibration are required to evaluate its potential clinical deployment.

## Introduction

Open tibial fractures, particularly those resulting from high-energy trauma, present a significant clinical challenge due to their high morbidity and the potential for severe complications such as fracture-related infection (FRI) and non-union. The management of these fractures is complex and often debated, with no consensus on the optimal fixation method. The Taylor Spatial Frame (TSF) has been shown to achieve union in 80.4 % of cases, although it is associated with a 33 % reoperation rate and significant deterioration in patient-reported outcomes over time [1]. The Gustilo-Anderson classification remains a key tool in guiding treatment, with recent data suggesting that internal fixation methods generally yield better outcomes [2]. The economic burden of open tibial fractures is substantial, with initial hospitalization costs ranging widely and a significant portion of patients unable to return to work within a year [3]. Non-union, occurring in about 12 % of cases, is associated with increased healthcare utilization and costs, as well as a higher likelihood of additional fractures and prolonged opioid use [4]. Implementing structured treatment protocols, such as those based on the BOAST 4 guidelines, has been shown to significantly reduce the rates of FRI and reoperation due to non-union [5]. Despite advancements in management strategies, predicting outcomes such as union, osteomyelitis, and amputation remains challenging, particularly in severe cases like Gustilo IIIC fractures [6]. The high rates of FRI and non-union in type III open tibial shaft fractures treated with open reduction and internal fixation further underscore the healthcare burden associated with these injuries [7]. Overall, the management of open tibial fractures requires a multidisciplinary approach that balances the need for timely intervention with the optimization of both biological and biomechanical environments to promote healing and minimize complications [8], 9].

Existing risk prediction tools in healthcare often fall short due to several critical limitations. These issues compromise the accuracy and generalizability of the models, as many clinical prediction models for cardiovascular diseases exhibited poor discrimination and calibration when applied to different populations [10]. Furthermore, the lack of consistent outcome definitions and limited reporting on calibration and decision-utility further exacerbate these shortcomings [11]. Landmarking involves creating models that use only the information available up to specific decision points, such as admission or postoperative day 3, which can enhance the model’s relevance and applicability in clinical settings [12]. This approach is supported by the development of interpretable models, such as the MARKED multi-label model, which predicts multiple postoperative complications simultaneously and provides a quantitative basis for complication management [13]. Additionally, the call for greater transparency and the use of explainable AI frameworks in risk prediction tools further emphasizes the need for models that are not only accurate but also interpretable and adaptable to evolving clinical practices [14]. By addressing these issues, landmarked strategies can improve the utility and reliability of risk prediction tools, ultimately enhancing patient outcomes and clinical decision-making processes.

Accordingly, the objective of this study was to develop and internally validate two leakage-robust, landmarked prediction models for 12-month FRI after open tibial fracture, anchored to two clinically meaningful decision points, admission and postoperative day 3 (POD3). We further aimed to quantify the incremental value of POD3 updating over admission-only prediction by comparing discrimination, calibration, and clinical utility using decision-curve analysis.

## Methods

### Study design and data source

We conducted a single-center retrospective cohort study at Tianjin Hospital from January 2023 to June 2024 to develop and internally validate two leakage-robust, landmarked prediction models for 12-month fracture-related infection (FRI) after open tibial fracture, with prespecified landmarks at admission and POD3. Data were abstracted from the electronic medical record and operative/anesthesia systems, with timestamps used to enforce landmark-specific availability of predictors. This study was conducted in accordance with the Declaration of Helsinki, as revised in 2013. The study protocol was reviewed and approved by the Ethics Committee of Tianjin Hospital (approval No. 2026084; approval date: June 20, 2025), which waived the requirement for informed consent because this was a retrospective analysis of de-identified routinely collected data and posed no more than minimal risk to participants. All data were anonymized prior to analysis.

### Participants

Eligible participants were patients aged ≥18 years with an open tibial fracture managed with limb salvage at the study center. The analytic dataset was restricted to fracture episodes considered at risk for developing 12-month FRI (e.g., excluding primary amputation prior to risk accrual and episodes without sufficient documentation to ascertain 12-month infection status). The unit of analysis was one fracture episode per patient. When bilateral injuries occurred, the first chronologic episode was retained to preserve independence.

Follow-up for the binary 12-month endpoint was defined from the index admission for the fracture episode until 12 months. Outcome surveillance used readmissions, reoperations, microbiology/pathology results, antibiotic courses, and outpatient assessments documented within the interval.

### Outcome definition and adjudication (12-month FRI)

The primary outcome was definite FRI occurring within 12 months, adjudicated using the international FRI consensus definition [15]. Definite FRI required ≥1 confirmatory criterion, including sinus/fistula or wound breakdown communicating with bone/implant, purulent drainage/pus observed clinically or intra-operatively, phenotypically indistinguishable pathogens isolated from ≥2 separate deep tissue/implant specimens, and/or microorganisms demonstrated in deep tissue by histopathology. Suggestive features such as nonspecific imaging changes, isolated elevation of inflammatory markers, or a single positive culture were not considered diagnostic in isolation and prompted additional chart review for confirmatory evidence. The 12-month window was the prespecified prediction horizon and does not redefine the underlying diagnostic framework. Two orthopedic trauma reviewers adjudicated FRI status using a standardized abstraction form, with disagreements resolved by a senior reviewer. Adjudicators were blinded to the calculated model risk scores and the 15 % threshold classification, although complete blinding to clinical course was not possible in retrospective chart review.

### Landmark definitions and predictor measurement windows

Two landmarks were prespecified: the admission landmark at the time of first IV antibiotic administration following presentation. “Time to first IV antibiotics” was operationalized as the interval (hours) from presentation to first IV antibiotic administration and is therefore fully observed at the admission landmark. POD3 landmark was defined as postoperative day 3 after the index operative management for the fracture episode. “Presentation” was defined as arrival time at the study center. For transferred patients, time-zero was arrival at our center; transfer status was retained as a baseline characteristic and used for interpretation of process-of-care intervals. Because the admission landmark is defined at first IV antibiotic administration, the admission model is intended for use at that decision point (after initial assessment), and predictors were restricted to information available up to that time. For the POD3 analysis, the landmark cohort included episodes that were alive, event-free, and still at risk at POD3; predictors were restricted to information accrued before or on POD3, and 12-month FRI status was assessed after the landmark within the prespecified 12-month horizon.

Candidate predictors were prespecified based on clinical plausibility and bedside availability and were restricted to variables observable up to each landmark to prevent information leakage. For the admission model, predictors included provisional emergency department Gustilo grade, contamination category, injury mechanism, baseline physiology, and time from presentation to first IV antibiotics, along with baseline host factors where available (e.g., diabetes and smoking status). For the POD3 model, predictors additionally included early clinical course variables observed through POD3, laboratory measures obtained during POD0–POD3, and early reconstruction metrics, including coverage achieved by day 3 and “days without coverage,” defined as the cumulative number of days without definitive soft-tissue coverage truncated at the POD3 landmark; time to first debridement was treated as a POD3-available process variable because it is not knowable at admission. Provisional emergency-department Gustilo grade was intentionally retained for the admission model because it reflects the information available at the admission decision point; definitive intraoperative grading was not substituted into the admission model to avoid post-landmark leakage. To reduce treatment-paradox and reverse-causation concerns, treatment and process variables were included only if observed before the corresponding landmark.

### Laboratory assays

Routine clinical assays were used, including complete blood count indices and C-reactive protein (CRP). To reduce reliance on arbitrary cut-offs in the setting of postoperative inflammation, laboratory predictors were modelled continuously. For POD3 prediction, laboratory predictors used the most recent value available prior to POD3 and, when available, an early within-hospital kinetic term to capture short-term changes.

### Missing data

Missing predictor data were handled using multiple imputation by chained equations under a missing-at-random assumption, performed separately within each landmark dataset to respect time dependent availability. We generated m=20 imputed datasets with 20 iterations per dataset; the imputation model included all candidate predictors, the outcome indicator, and auxiliary timestamp variables (e.g., time to antibiotics/debridement) to improve plausibility of the missing-at-random assumption. Continuous variables were imputed using predictive mean matching and categorical variables using logistic or multinomial regression. To avoid optimistic validation, imputation was nested within the bootstrap procedure such that each resample underwent its own imputation, model fitting, and evaluation, consistent with the prespecified internal validation strategy. Before imputation, we summarized missingness by candidate predictor and by outcome group. As sensitivity analyses for the missing-at-random assumption, we repeated model evaluation using complete cases and a delta-adjusted imputation scenario in which imputed process delays and inflammatory markers were shifted by 0.25 SD toward the higher-risk direction before prediction.

### Model development

For each landmark, we fit an elastic-net penalized logistic regression model to estimate absolute risk of 12-month FRI, using restricted cubic splines for continuous predictors to accommodate nonlinearity while avoiding categorization. Continuous predictors were centered/scaled prior to penalization, spline complexity was kept conservative, and elastic-net tuning was performed within resampling to prioritize shrinkage and stability in the presence of correlated predictors. Model complexity was prespecified to remain compatible with the observed event count by limiting the effective number of predictor parameters. Elastic-net mixing parameter α was fixed at 0.5 in the primary analysis, and λ was selected by 10-fold cross-validation minimizing mean binomial deviance within each bootstrap resample; the final reported models used α=0.5 and λ=0.012 (admission) and λ=0.010 (POD3) when refit on the full landmark datasets. To facilitate reproducibility and clinical adoption, the retained shrunken coefficients and coding/centering notes for both final refit models are reported, consistent with transparent reporting recommendations for prediction models [16].

### Internal validation, performance, and clinical utility

Internal validation used 1,000 bootstrap resamples with optimism correction, repeating the full modeling pipeline (including nested imputation and penalization tuning) within each resample. When comparing admission and POD3 models in decision curve analysis, we applied both models to the POD3 landmark cohort so that net benefit curves were generated on the same at-risk population. Bootstrap resamples were used for optimism correction and performance estimation; the final reported model coefficients were obtained by refitting on the full landmark datasets using the prespecified tuning procedure.

Discrimination was summarized by area under the ROC curve (AUC) and overall accuracy by Brier score. Calibration was evaluated using calibration-in-the-large (intercept), calibration slope, and calibration plots comparing observed vs. predicted risks across the prediction range. Clinical utility was assessed using decision curve analysis across threshold probabilities from 5 to 25 %, comparing model net benefit against “treat all” and “treat none” strategies.

### Statistical analysis

Baseline characteristics were summarized overall and by 12-month FRI status using mean ± SD or median [IQR] for continuous variables and n (%) for categorical variables. Descriptive p values were calculated using t-test or Wilcoxon rank-sum for continuous variables and χ^2^ or Fisher’s exact test for categorical variables, with Benjamini–Hochberg false discovery rate (FDR) adjustment. Thes p value were retained as descriptive summaries of baseline imbalance and were not used for predictor screening, variable selection, or model tuning.

For prespecified clinical stratification, the admission model was evaluated at a 15 % predicted-risk threshold, reporting the confusion matrix and operating characteristics (sensitivity, specificity, PPV, NPV) and enrichment metrics (risk difference, risk ratio, odds ratio) with 95 % confidence intervals (CIs). Wilson intervals were used for single proportions; Newcombe-type confidence intervals were used for risk differences. Log-scale Wald confidence intervals were used for risk ratios and odds ratios, and Fisher’s exact test was used for group-comparison p values.

We additionally performed a multi-threshold clinical impact analysis across prespecified thresholds of 5 %, 10 %, 15 %, 20 %, and 25 %, reporting sensitivity, specificity, TP/FP/FN/TN, PPV/NPV, and the number of patients flagged per FRI captured. To address missed infections at the 15 % threshold, we performed a descriptive subgroup analysis comparing infected episodes classified as false negatives vs. true positives.

## Results

### Study population and 12-month FRI incidence

**Figure 1:**
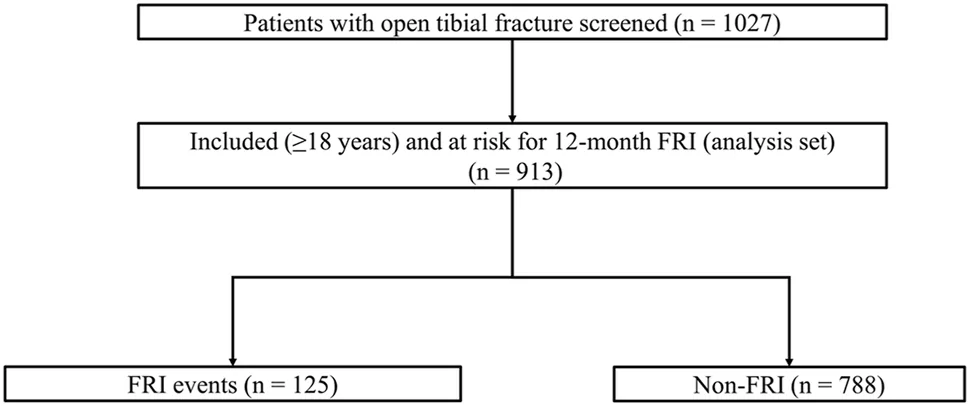
Cohort flow and landmark design. Flow diagram summarizing the analysis population (n=913; 12-month FRI n=125, 13.7 %) and the two prespecified landmarks (admission; postoperative day 3) .

The at-risk analytic cohort included 913 eligible open tibial fracture episodes (Figure 1), among which 125 developed definite FRI within 12 months, corresponding to an incidence of 13.7 % (125/913). This cohort included 274/913 (30.0 %) transfer-in episodes and a high proportion of provisional Gustilo-Anderson type IIIB/IIIC injuries (475/913, 52.0 %), reflecting the referral case-mix (Table 1).

**Table 1:** Baseline patient and injury characteristics.

Characteristic	FRI (n=125)	No. FRI (n=788)	p-Value	q-Value, FDR
Age (years), mean ± SD	44.3 ± 13.3	41.4 ± 15.0	0.0306	0.0421
Male, n (%)	95/125 (76.0)	581/788 (73.7)	0.669	0.669
Diabetes, n (%)	18/125 (14.4)	64/788 (8.1)	0.0347	0.0424
Current smoker, n (%)	51/125 (40.8)	241/788 (30.6)	0.0299	0.0421
High-energy mechanism, n (%)	98/125 (78.4)	514/788 (65.2)	0.00499	0.0110
Provisional ED gustilo, n (%)			2.74e-08	3.02e-07
II	13 (10.4)	188 (23.9)		
III A	18 (14.4)	219 (27.8)		
III B	64 (51.2)	301 (38.2)		
III C	30 (24.0)	80 (10.2)		
Contamination, n (%)			0.0157	0.0288
None/low	55 (44.0)	447 (56.7)		
Moderate	43 (34.4)	231 (29.3)		
High	27 (21.6)	110 (14.0)		
Transfer-in, n (%)	45/125 (36.0)	229/788 (29.1)	0.142	0.156
Time to first IV antibiotics (h), median [IQR]	1.71 [0.98, 2.95]	1.14 [0.66, 1.94]	5.64e-07	3.10e-06
Time to first debridement (h), median [IQR]	13.22 [9.03, 17.56]	10.05 [7.23, 14.17]	2.79e-06	1.02e-05
Definitive soft-tissue coverage ≤72 h, n (%)	65/125 (52.0)	529/788 (67.1)	0.00140	0.00384

p-Values and FDR-adjusted q-Values are descriptive only and were not used for predictor selection, penalization tuning, or model specification.

### Baseline characteristics

Patient, injury, and early care-process characteristics for the overall cohort and stratified by 12-month FRI status are presented in Table 1, with descriptive hypothesis tests reported alongside Benjamini–Hochberg FDR-adjusted q values to account for multiplicity (Table 1). Missingness was concentrated in process-of-care and POD0-POD3 laboratory variables; no candidate predictor exceeded 12.4 % missingness, and 12-month outcome status was complete for all 913 analytic episodes (Supplementary Table S1A). Complete-case and delta-adjusted imputation sensitivity analyses produced similar discrimination and overall accuracy to the primary multiple-imputation analysis: admission AUC ranged from 0.76 to 0.77 and POD3 AUC ranged from 0.81 to 0.82 (Supplementary Table S1B).

### Admission landmark model performance

**Figure 2:**
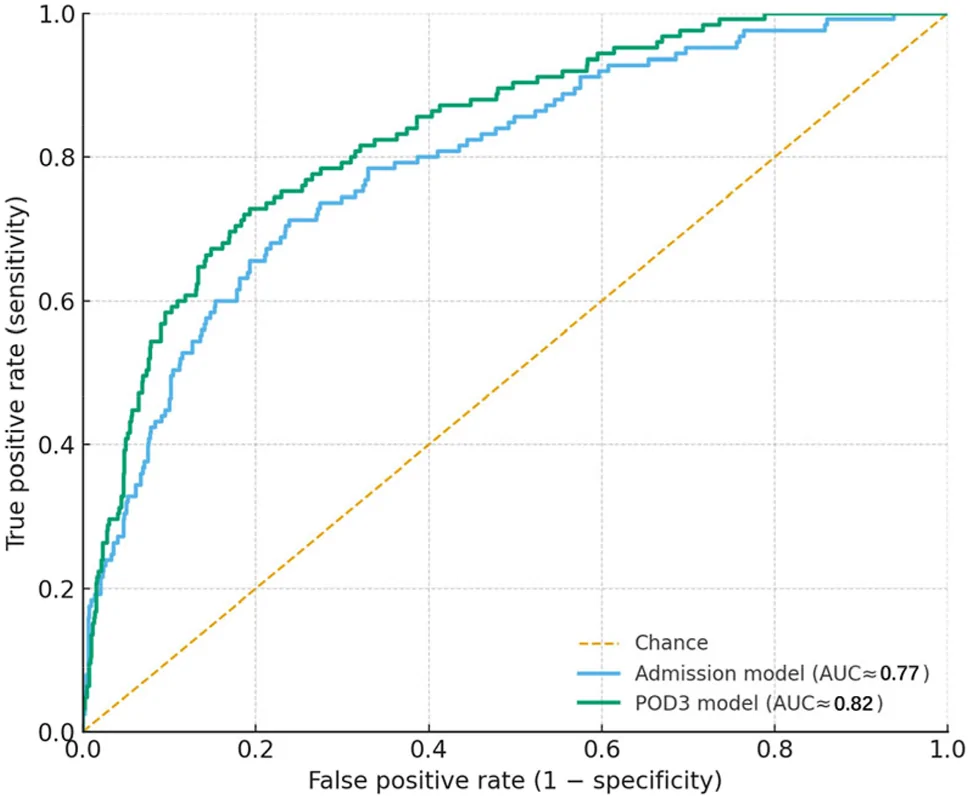
ROC curves. Receiver operating characteristic (ROC) curves illustrating the reported discrimination for the admission model (AUC 0.77) and POD3 model (AUC 0.82).

After optimism correction using 1,000 bootstrap resamples with nested imputation, the admission landmark model demonstrated moderate discrimination with an optimism-corrected AUC of 0.77 and good overall accuracy with a Brier score of 0.097 (Table 2 and Figure 2).

**Table 2:** Internal validation performance (optimism-corrected).

Metric	Admission landmark model	POD3 landmark model
Sample size used for model, n	913	880
FRI events, n (%)	125 (13.7)	118 (13.4)
AUC (95 % CI, optimism-corrected)	0.77 (0.72–0.82)	0.82 (0.77–0.87)
Brier score (95 % CI, optimism-corrected)	0.097 (0.086–0.109)	0.089 (0.079–0.100)
Calibration intercept (95 % CI)	−0.03 (−0.20 to 0.14)	−0.02 (−0.18 to 0.14)
Calibration slope (95 % CI, optimism-corrected)	0.97 (0.82–1.13)	1.02 (0.86–1.18)
Brier skill score vs. null	0.179	0.247

**Figure 3:**
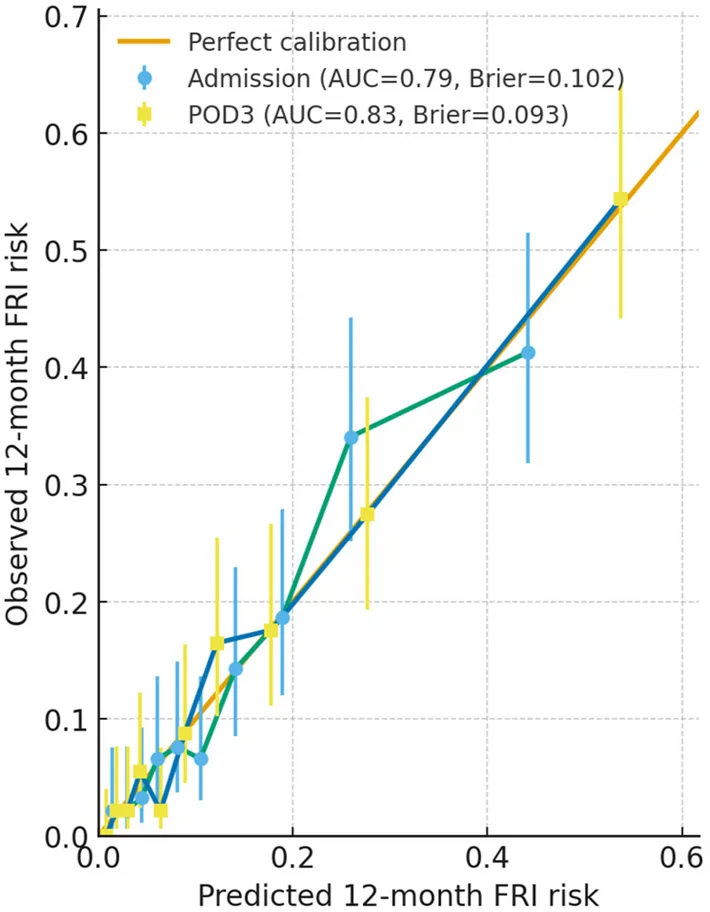
Calibration plot. Calibration with the 45° line representing perfect calibration.

Calibration was assessed using calibration slope/intercept and visual calibration plots to compare predicted and observed 12-month risks across the prediction range (Table 2 and Figure 3).

### Risk stratification at the prespecified 15 % threshold (admission model)

At the prespecified 15 % predicted-risk threshold, 112/913 episodes (12.3 %) were classified as high risk and had a substantially higher observed FRI rate than those classified as lower risk (28.6 % [32/112] vs. 11.6 % [93/801]), capturing 25.6 % of all FRIs (32/125) while maintaining 89.8 % specificity (708/788) (Table 3). This corresponded to a risk difference of 17.0 % (95 % CI 7.0–28.0), a risk ratio of 2.46 (95 % CI 1.73–3.49), and an odds ratio of 3.05 (95 % CI 1.92–4.84), with Fisher’s exact p=1.2×10^−5^ (q=1.2×10^−5^) for the event-rate difference between predicted groups (Table 3). Operating characteristics at this threshold were sensitivity 25.6 % and negative predictive value 88.4 %, reflecting meaningful risk enrichment in a relatively small, flagged subgroup (Table 3). Sensitivity at this operating point was 25.6 %, indicating that this cut-off functions as a high specificity enrichment threshold.

**Table 3:** Admission model classification at 15 % risk threshold.

A. Confusion matrix (threshold=15 %)			
Predicted group	Observed FRI, n	Observed no FRI, n	Total n, %
High risk (≥15 %)	32	80	112 (12.3)
Lower risk (<15 %)	93	708	801 (87.7)
Total	125	788	913 (100.0)

B. Diagnostic/clinical utility metrics				
Metric	Estimate	95 % CI	p-Value	q-Value, FDR
High-risk FRI rate, %	28.6	21.0–37.5	–	–
Lower-risk FRI rate, %	11.6	9.6–14.0	–	–
Risk difference, %	17.0	7.0–28.0	1.2 × 10^−5^	1.2 × 10^−5^
Risk ratio	2.46	1.73–3.49	1.2 × 10^−5^	1.2 × 10^−5^
Odds ratio	3.05	1.92–4.84	1.2 × 10^−5^	1.2 × 10^−5^
Sensitivity, %	25.6	18.8–33.9	–	–
Specificity, %	89.8	87.5–91.8	–	–
PPV, %	28.6	21.0–37.5	–	–
NPV, %	88.4	86.0–90.4	–	–

### Multi-threshold clinical impact analysis

Across risk thresholds from 5 to 25 %, lowering the threshold increased sensitivity at the cost of substantially more patients being classified high risk (Table 4). For example, sensitivity increased from 25.6 % at 15% to 44.8 % at 10 % (with 27.4 % of patients flagged) and to 75.2 % at 5 % (with 60.2 % of patients flagged). Conversely, higher thresholds improved specificity and PPV but further reduced sensitivity (Table 4).

**Table 4:** Multi-threshold clinical impact analysis of the admission model.

Threshold, %	High risk n, %	TP	FP	FN	TN	Sensitivity, %	Specificity, %	PPV, %	NPV, %	Flagged per FRI captured
5	550 (60.2)	94	456	31	332	75.2	42.1	17.1	91.5	5.9
10	250 (27.4)	56	194	69	594	44.8	75.4	22.4	89.6	4.5
15	112 (12.3)	32	80	93	708	25.6	89.8	28.6	88.4	3.5
20	70 (7.7)	22	48	103	740	17.6	93.9	31.4	87.8	3.2
25	45 (4.9)	16	29	109	759	12.8	96.3	35.6	87.4	2.8

### Descriptive analysis of missed infections at the 15 % threshold

Among the 125 infections, 93/125 (74.4 %) were false negatives at the 15 % admission threshold. Compared with true positives (n=32), false negatives had a lower proportion of Gustilo IIIC injuries and high contamination, but still included a substantial number of severe IIIB/IIIC injuries, underscoring that many infections occur outside the highest predicted-risk stratum (Table 5).

**Table 5:** Descriptive characteristics of infected cases missed vs. detected at the 15 % admission threshold.

Variable	True positives (infected & flagged, n (%)	False negatives (infected & missed, n (%)
Diabetes mellitus	5 (15.6)	13 (14.0)
Current smoker	14 (43.8)	37 (39.8)
High-energy mechanism	28 (87.5)	70 (75.3)
Transfer-in to study center	14 (43.8)	31 (33.3)
Provisional gustilo grade: II	1 (3.1)	12 (12.9)
Provisional gustilo grade: III A	3 (9.4)	15 (16.1)
Provisional gustilo grade: III B	16 (50.0)	48 (51.6)
Provisional gustilo grade: III C	12 (37.5)	18 (19.4)
Contamination: None/low	10 (31.2)	45 (48.4)
Contamination: Moderate	12 (37.5)	31 (33.3)
Contamination: High	10 (31.2)	17 (18.3)
Definitive coverage ≤72 h	13 (40.6)	52 (55.9)
Time to first IV antibiotics, h (median [IQR])	2.20 [1.30, 3.60]	1.55 [0.90, 2.70]
Time to first debridement, h (median [IQR])	14.6 [10.2, 18.5]	12.7 [8.8, 17.0]

### POD3 landmark model performance and clinical utility

In the POD3 landmark cohort (event-free and at risk at POD3), model updating modestly improved discrimination and accuracy relative to admission, achieving an optimism-corrected AUC of 0.82 and Brier score of 0.089 (Table 2). The absolute AUC increase vs. admission was 0.05.

**Figure 4:**
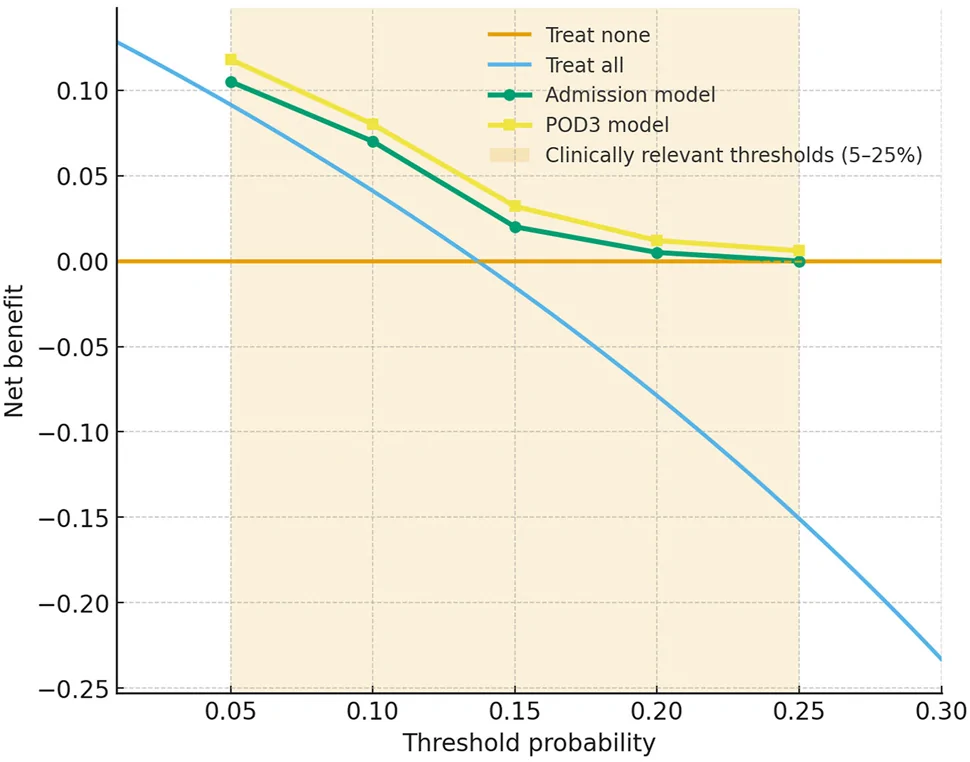
Decision curve analysis. Decision curve showing “treat none” (net benefit=0) and “treat all” (computed from prevalence 13.7 %) across threshold probabilities. The shaded region highlights the threshold range (5–25 %).

Decision curve analysis indicated slightly higher net benefit for the POD3 model across threshold probabilities from 5 to 25 % compared with the admission model and default “treat all” and “treat none” strategies (Figure 4), supporting incremental clinical utility of POD3 updating within the prespecified threshold range. The absolute net benefit gain for POD3 vs. admission was 0.003–0.006 across the prespecified thresholds, with 95 % CI compatible with small gains at some thresholds (Table 6). For transparency, the retained refit coefficients and implementation notes for the admission and POD3 models are provided in Supplementary Table S2.

**Table 6:** Numerical decision-curve results across prespecified clinical thresholds.

Threshold probability	Admission net benefit	POD3 net benefit	POD3 – admission (95 % CI)	Treat-all net benefit
5 %	0.101	0.107	0.006 (−0.001 to 0.013)	0.088
10 %	0.071	0.077	0.006 (0.000–0.013)	0.038
15 %	0.022	0.028	0.006 (0.001–0.012)	−0.019
20 %	0.006	0.011	0.005 (−0.001 to 0.011)	−0.083
25 %	0.001	0.004	0.003 (−0.002 to 0.008)	−0.155

Net benefit was calculated on the POD3 at-risk cohort for paired comparison of the admission and POD3 models; treat-none net benefit is 0 at all thresholds.

## Discussion

Our principal finding is that two prespecified, leakage-robust landmark models produced clinically interpretable within the derivation setting, internally validated estimates of 12-month fracture-related infection risk after open tibial fracture, with a modest incremental improvement when updating predictions at postoperative day 3. The admission model achieved moderate discrimination and good overall accuracy and provided actionable risk stratification. Updating at POD3, using only information accrued through day 3 modestly improved performance and demonstrated slightly higher net benefit across clinically relevant thresholds, indicating that short-term postoperative evolution contains additional prognostic signal beyond admission data. However, the absolute discrimination gain was small (AUC +0.05), decision-curve separation was modest, and at the prespecified 15 % admission threshold sensitivity was 25.6 %, emphasizing risk enrichment rather than a high-sensitivity screening/triage tool. Consistent with the dominant role of injury severity and contamination at presentation, these findings suggest that a substantial portion of FRI risk is already apparent at admission, with POD3 updating providing incremental refinement rather than overturning admission-based prognosis. Together, these results support a pragmatic two-step workflow, admission risk enrichment at admission followed by POD3 recalibration, while underscoring the need for external validation before deployment.

Mechanistically, the strongest pathways map cleanly onto established FRI biology. Injury severity and contamination proxy the combined burden of inoculum, devascularization, and soft-tissue destruction first formalized in classic open-fracture work and reiterated in contemporary open tibial FRI reviews [17], 18]. These upstream drivers create the permissive environment for persistent infection around devitalized tissue and fixation, aligning with consensus principles that prioritize adequate debridement, stability, dead-space control, and timely soft-tissue coverage to prevent and manage FRI after open tibial fracture [15], 19]. Time-sensitive care processes further fit bacterial kinetic logic. BOAST recommends IV antibiotics as soon as possible (ideally within 1 h) [20], historical evidence identifies early antibiotics as a key modifiable determinant of infection risk, and type III open tibia data show lower infection rates with immediate prophylaxis [21]. While the evidence does not support a universal rigid “6-h debridement rule,” meta-analytic data support interpreting increasing delay as plausibly harmful, consistent with modeling delay continuously rather than as a single cut-off [22]. Early definitive coverage similarly functions as an “exposure time” construct. Early reconstruction paradigms and orthoplastic series (“fix-and-flap”) show better outcomes when coverage is achieved promptly, with delays beyond ∼72 h often associated with complications, mirroring orthoplastic standards for open fractures [20], 23], 24].

The modest performance gain of the POD3 update together with slightly higher net benefit across 5–25 % thresholds support the interpretation that early postoperative course variables and laboratories operate as integrators of multiple latent processes (residual contamination, adequacy of debridement, evolving ischemia/necrosis, and coverage status) into a single “host–wound state” signal for FRI after open tibial fracture.

At the same time, serum inflammatory markers should not be framed as stand-alone diagnostics. A systematic review/meta-analysis shows that CRP/WBC/ESR are insufficiently accurate to diagnose late FRI in isolation [25]. This is consistent with the FRI consensus framework, which emphasizes confirmatory clinical, microbiologic, and histopathologic evidence rather than nonspecific systemic inflammation [15], 19]. Therefore, it is biologically and methodologically defensible that POD3 laboratories (and their trajectories) enhance risk stratification when combined with injury severity, contamination, and early management variables, precisely the clinical reality in which clinicians reassess the wound trajectory after initial surgical care in open tibial fracture patients.

Clinically, the models offer two realistic decision points for an IF≈3 trauma/orthopedics audience. At admission, an illustrative 15 % risk threshold identified 112/913 (12.3 %) patients as high risk with an observed FRI rate of 28.6 vs. 11.6 % in lower-risk patients, capturing ∼26 % of all FRIs, which can be used to prioritize early orthoclastic coordination, heightened monitoring, and selective early infectious diseases/microbiology involvement in open tibial fracture care pathways, consistent with BOAST’s emphasis on specialist orthoplastic management and rapid antibiotics [20] and with FRI consensus recommendations for multidisciplinary management and antimicrobial strategy [19], 26]. However, sensitivity at this operating point was 25.6 % (i.e., 74.4 % of infections were not flagged), so this cut-off should not be used as a rule-out or de-escalation tool for “lower-risk” patients; rather, it identifies a small subgroup with enriched risk for targeted escalation.

The POD3 update then provides a second checkpoint to refine follow-up intensity, activate escalation pathways, and enrich enrolment into prevention studies, an important consideration given that large pragmatic open-fracture trials show that some commonly used interventions have nuanced benefits and may be most efficient when targeted to high-risk subgroups [27]. Finally, we intentionally avoid proposing a single “best” cut-off. Decision curve analysis was developed to compare strategies across ranges of threshold probabilities and explicitly reflects local preferences and consequences of false positives vs. missed infections, so thresholds should be adapted to resources, baseline risk, and patient values rather than treated as universal constants [28], 29].

Several important limitations remain in our study. The single-center retrospective design may embed local practice patterns that affect transportability, provisional emergency-department Gustilo grading is susceptible to measurement error, missing data and unmeasured confounding are unavoidable in retrospective records, and a potential “treatment paradox” (higher-risk patients receiving more aggressive prevention) could attenuate associations between predictors and FRI. In addition, our center’s referral case-mix included a high proportion of severe Gustilo IIIB/IIIC injuries and transfers, which may limit generalizability and lead to miscalibration if the model is applied in lower-risk trauma populations without recalibration. Regarding treatment paradox, because prophylactic intensity is partly driven by perceived risk, predictor–outcome associations may be biased toward the null; the models should therefore be interpreted as predictive tools rather than causal effect estimators. We mitigated these risks by prespecifying landmarks and predictor windows, using shrinkage and optimism correction, and emphasizing calibration and clinical utility rather than causal interpretation. Finally, because the models are internally validated only, external validation in multicenter cohorts will be essential before clinical deployment, followed by a prospective impact study to test whether implementing admission/POD3 risk estimates improves outcomes or resource utilization in FRI after open tibial fracture. Outcome adjudicators were blinded to model risk scores, but complete blinding to clinical course was not feasible because confirmatory FRI criteria are embedded in routine clinical documentation. The single-center case-mix and referral pattern represent an applicability limitation for prediction models [30]. Missing-data sensitivity analyses were directionally consistent with the primary analysis, but they cannot prove the missing-at-random assumption.

In conclusion, we developed two prespecified, leakage-robust landmark prediction models that provide clinically interpretable estimates of 12-month FRI risk after open tibial fracture at two real decision points, admission and POD3, using only information available up to each landmark. These findings support a pragmatic two-step workflow of admission risk enrichment followed by POD3 recalibration, while emphasizing that POD3 updating offers modest incremental refinement and that external validation and potential recalibration are required before broad clinical deployment for risk enrichment.

## Supplementary Material

Supplementary Material
