# Supplementary material for: Predictors of surgical site infection/fracture related infection after open tibial fracture
Source: Open Med (Wars). 2026 Sep 2;21(1):20261493. doi: 10.1515/med-2026-1493 (PMC13533289; doi:10.1515/med-2026-1493)
Supplement: Supplementary file 1 [file j_med-2026-1493_suppl_001.docx]

**Supplementary Table S1. Missing data and sensitivity analyses**

**A. Missingness by candidate predictor**

| **Variable** | **Landmark dataset** | **Denominator** | **Missing n (%)** |
| --- | --- | --- | --- |
| **12-month FRI outcome status** | Admission | 913 | 0 (0.0) |
| **Diabetes mellitus** | Admission | 913 | 8 (0.9) |
| **Current smoker** | Admission | 913 | 31 (3.4) |
| **Contamination category** | Admission | 913 | 18 (2.0) |
| **Time to first IV antibiotics** | Admission | 913 | 43 (4.7) |
| **Time to first debridement** | POD3 | 880 | 51 (5.8) |
| **Definitive coverage <=72 h** | POD3 | 880 | 26 (3.0) |
| **WBC during POD0-POD3** | POD3 | 880 | 42 (4.8) |
| **CRP during POD0-POD3** | POD3 | 880 | 109 (12.4) |

**B. Sensitivity analyses for missing data**

| **Analysis** | **Landmark** | **n/events** | **AUC (95% CI)** | **Brier score (95% CI)** | **Calibration slope (95% CI)** |
| --- | --- | --- | --- | --- | --- |
| **Primary multiple imputation** | Admission | 913/125 | 0.77 (0.72-0.82) | 0.097 (0.086-0.109) | 0.97 (0.82-1.13) |
| **Complete-case analysis** | Admission | 834/111 | 0.76 (0.71-0.81) | 0.100 (0.088-0.113) | 0.94 (0.78-1.11) |
| **Delta-adjusted imputation** | Admission | 913/125 | 0.76 (0.71-0.82) | 0.099 (0.087-0.111) | 0.95 (0.80-1.11) |
| **Primary multiple imputation** | POD3 | 880/118 | 0.82 (0.77-0.87) | 0.089 (0.079-0.100) | 1.02 (0.86-1.18) |
| **Complete-case analysis** | POD3 | 748/100 | 0.81 (0.76-0.86) | 0.091 (0.080-0.103) | 0.99 (0.82-1.17) |
| **Delta-adjusted imputation** | POD3 | 880/118 | 0.81 (0.76-0.86) | 0.090 (0.080-0.102) | 1.00 (0.84-1.16) |

*Delta-adjusted imputation shifted imputed process-delay and inflammatory-marker values by 0.25 SD toward the higher-risk direction before prediction; values are reported as sensitivity analyses, not separate model-development procedures.*

**Supplementary Table S2. Retained final refit model coefficients for implementation**

| **Model** | **Predictor/coding** | **Coefficient (β)** | **OR (95% CI)** |
| --- | --- | --- | --- |
| **Admission** | Intercept | -3.05 | — |
| **Admission** | Age, per 10 years | 0.08 | 1.08 (0.94-1.25) |
| **Admission** | Diabetes mellitus | 0.42 | 1.52 (0.92-2.51) |
| **Admission** | Current smoker | 0.28 | 1.32 (0.91-1.93) |
| **Admission** | High-energy mechanism | 0.39 | 1.48 (1.00-2.20) |
| **Admission** | Gustilo IIIA | 0.44 | 1.55 (0.84-2.86) |
| **Admission** | Gustilo IIIB | 0.87 | 2.39 (1.40-4.08) |
| **Admission** | Gustilo IIIC | 1.35 | 3.86 (2.05-7.27) |
| **Admission** | Moderate contamination | 0.35 | 1.42 (0.95-2.13) |
| **Admission** | High contamination | 0.62 | 1.86 (1.11-3.12) |
| **Admission** | Transfer-in | 0.16 | 1.17 (0.80-1.72) |
| **Admission** | Time to first IV antibiotics, per h | 0.20 | 1.22 (1.08-1.39) |
| **POD3** | Intercept | -3.22 | — |
| **POD3** | Age, per 10 years | 0.07 | 1.07 (0.92-1.24) |
| **POD3** | Diabetes mellitus | 0.35 | 1.42 (0.84-2.39) |
| **POD3** | Current smoker | 0.21 | 1.23 (0.83-1.83) |
| **POD3** | High-energy mechanism | 0.32 | 1.38 (0.91-2.09) |
| **POD3** | Gustilo IIIA | 0.34 | 1.40 (0.75-2.63) |
| **POD3** | Gustilo IIIB | 0.72 | 2.05 (1.18-3.57) |
| **POD3** | Gustilo IIIC | 1.12 | 3.06 (1.58-5.92) |
| **POD3** | Moderate contamination | 0.30 | 1.35 (0.88-2.07) |
| **POD3** | High contamination | 0.50 | 1.65 (0.97-2.81) |
| **POD3** | Time to first IV antibiotics, per h | 0.14 | 1.15 (1.00-1.32) |
| **POD3** | Time to first debridement, per 6 h | 0.22 | 1.25 (1.05-1.48) |
| **POD3** | No definitive coverage by POD3 | 0.58 | 1.79 (1.17-2.75) |
| **POD3** | Days without coverage through POD3, per day | 0.28 | 1.32 (1.08-1.62) |
| **POD3** | Most recent CRP, per 50 mg/L | 0.18 | 1.20 (1.02-1.42) |
| **POD3** | CRP change, per 50 mg/L | 0.15 | 1.16 (0.99-1.37) |
| **POD3** | Most recent WBC, per 5 x 10^9^/L | 0.10 | 1.11 (0.92-1.34) |

*Risk is calculated as p = 1/[1 + exp(-linear predictor)]. Coefficients are shrunken final-refit coefficients. Odds ratios are shown for interpretability.*
